# A novel homeotic mutation in morphology using skeletal specimens in four breeds of Japanese indigenous chickens

**DOI:** 10.1016/j.psj.2026.107475

**Published:** 2026-07-21

**Authors:** Yume Okada, Yuma Nishida, Dipson Gyawali, Prudence Nyirimana, Daisuke Kondoh, Ryota Iwasaki, Jumpei Tomiyasu, Masaki Eda, Tatsuhiko Goto

**Affiliations:** a Department of Life and Food Sciences, Obihiro University of Agriculture and Veterinary Medicine, Obihiro, Hokkaido, 080-8555, Japan; b Department of Veterinary Medicine, Obihiro University of Agriculture and Veterinary Medicine, Obihiro, Hokkaido, 080-8555, Japan; c Hokkaido University Museum, Hokkaido University, Sapporo, Hokkaido, 060-0810, Japan; d Research Center for Global Agromedicine, Obihiro University of Agriculture and Veterinary Medicine, Obihiro, Hokkaido, 080-8555, Japan

**Keywords:** Breed difference, Chicken, Rib bone, Morphology

## Abstract

Four Japanese indigenous chicken breeds (Tosa-jidori, Ko-shamo, Minohikichabo, and Chabo) indicate morphological diversity, which is supported by their separate phylogenetic positions. These resources have high potential to be a unique model in morphological genetics. Therefore, this study aims to find morphological characteristics using skeletal specimens and computed tomography images at 7 weeks of age and the adult stage. The Tosa-jidori had significantly higher body weight and body length than the others, whereas Chabo showed a significantly lower ratio of neck length to body length than the others. Our observation identified novel mutants (individuals with eight ribs) across the four Japanese indigenous breeds. Significant breed differences were observed in the ratio of wild-type (seven ribs) to mutant (eight ribs) at both 7 weeks of age and the adult stage. The wild-type was predominant in Tosa-jidori and Ko-shamo, whereas the mutant was the majority or half-and-half in Minohikichabo and Chabo. Based on the total number of cervical and thoracic vertebrae, the present eight ribs should represent a novel homeotic mutation. This study mentioned the morphological characteristics of four Japanese indigenous breeds and documented novel variation of rib number within an avian species, the chicken.

## Introduction

Chickens were primarily domesticated up to 3,500 years ago, from the red jungle fowl (*Gallus gallus*), which is the main wild ancestor of the chicken (Wang et al., 2020; Eda, 2021; Peters et al., 2022). Over the years, genetic diversity has been subsequently created in the chicken population. Until now, more than 500 chicken breeds in the world have been established through long-term breeding efforts. Of them, there are approximately 45 Japanese indigenous breeds of chicken in Japan (Tsudzuki, 2003; Matsuda et al., 2025). Therefore, Japan is known as a diverse hotspot of chicken breeds (Yonezawa et al., 2022). Since most Japanese indigenous breeds have been created for ornamental purposes, there is morphological diversity, especially in tail feathers and body shape. Chickens are used in both basic and applied science to understand the life phenomena in developmental, physiology, immunology, medical, nutritional, and genomics research (Cheng and Burt, 2018). Most of those research targets are commercial layer and broiler strains, including White Leghorn, Rhode Island Red, White Plymouth Rock, and White Cornish, with extensive breeding history in Europe and the United States. The majority of Japanese indigenous chicken breeds are mostly separated from the foreign industrial breeds, including the layer- and broiler-related breeds in a phylogenetic tree (Osman et al., 2006; Matsuda et al., 2025). Although the use of the Japanese indigenous breeds has a large potential to understand the genotype-phenotype relationship (Goto and Tsudzuki, 2017), the genetic resources are used less frequently in basic and applied research.

The indigenous chickens, which exhibit specific morphologies, represent a large part of the genetic diversity in chickens. Of the approximately 45 breeds of Japanese indigenous chickens, 15 breeds and two groups (Jidori and Shamo) have been designated as national “Natural Monuments of Japan” in recognition of their rarity and value as genetic resources (Tsudzuki, 2003). Since the main use of Japanese indigenous chickens is for ornamental purposes, their population sizes tend not to be large. The various morphological characteristics exhibited by Japanese indigenous breeds have been indicated mainly at the adult stage in photographs (Tsudzuki, 2003). Thus, phenotypic analysis of morphology during the growth stages in Japanese indigenous chickens is required for a deeper understanding. In order to obtain guidelines for the conservation and reproduction of rare Japanese indigenous chicken breeds, it is necessary to clarify genetic variability in growth traits, morphological traits, and genetic relatedness. The untapped genetic resources will have novel phenomena to understand the genetic basis of phenotypic diversity in morphological traits.

**Fig. 1:**
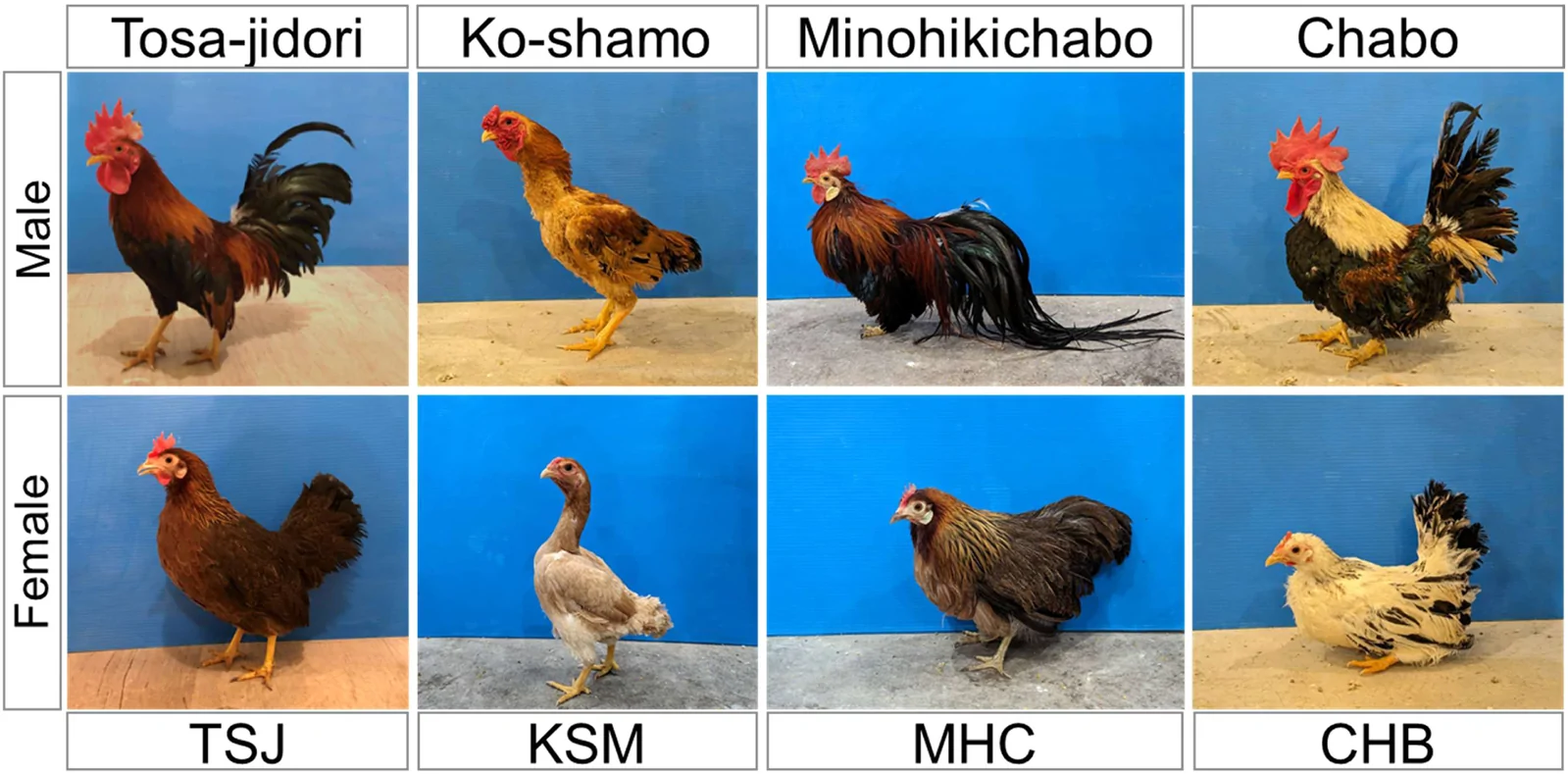
Photos of four Japanese indigenous breeds at the adult stage.

The body shape of chickens can be broadly divided into red jungle fowl (RJF)-type, Malay-type, Cochin-type, and others (Tsudzuki, 2003). We focused on four miniature Japanese indigenous chicken breeds, Tosa-jidori, Ko-shamo, Minohikichabo, and Chabo, which together cover approximately the full genetic diversity of the 45 Japanese indigenous breeds (Osman et al., 2006). Tosa-jidori and Minohikichabo exhibit the RJF-type of body shape. Ko-shamo is categorized as the Malay-type of body shape. Chabo exhibits a ball-like body shape (round body outline [Kudo et al., 2017]) that is not classified as any of the above. In addition, Tosa-jidori (Fig. 1) shows a wild-type appearance (such as plumage color and angle of tail feathers) and is similar to that of the RJF. Minohikichabo (Fig. 1) is characterized by short legs and rich tail feathers. Ko-shamo (Fig. 1), which is classified in the Shamo group, shows a walnut comb, an erect body, strong muscularity, and short tail feathers. Chabo (Fig. 1) is characterized by short legs and upright tail feathers. Since the pairwise genetic distance (*D_A_*) among the four breeds ranged from 0.352 to 0.506 (Osman et al., 2006), there are likely to be breed-specific genetic differences underlying the morphological diversity.

We previously conducted phenotypic analysis of morphological traits, including body weight, shank length, tail feather length, and number of tail feathers at 1-36 weeks of age for the three Japanese indigenous breeds, Tosa-jidori, Chabo, and Minohikichabo (Ono et al., 2022). We revealed significant breed differences that Tosa-jidori is heavier, Chabo and Minohikichabo have shorter shank length, Minohikichabo has rich tail feathers, and male Minohikichabo has significantly longer tail feathers (Ono et al., 2022). Kondoh et al. (2022) focused on the morphology of the tail parts, which have huge phenotypic diversity among the breeds, and identified significant breed differences in the skeletal morphology of the tail parts of the Tosa-jidori, Chabo, and Minohikichabo, with each of the three breeds having a significantly different breed-specific angle of the tailbone (pygostyle bone). In addition, analysis of the skeletal morphology of the tail parts using F_1_ hybrids produced by crosses between Tosa-jidori and Minohikichabo has revealed that various modes of inheritance control the length (full dominance), thickness (over-dominance), and angle of the tailbone (partial dominance) (Nyirimana et al., 2024). Kudo et al. (2016; 2017) have investigated the morphology of the skull, skeletal forelimb, pectoral girdle, and sternum in six Japanese indigenous breeds, including Chabo and Tosa-jidori. However, little research has been conducted on the whole-body skeleton in Tosa-jidori, Ko-shamo, Minohikichabo, and Chabo. By comparing the visual appearance of the four Japanese indigenous breeds shown in Fig. 1, we hypothesized that the neck length differs in each breed.

The bones of the chicken are broadly divided into the skull bones and cervical, thoracic, lumbar, sacral, and caudal vertebrae (Wellik, 2007). The cervical bones are defined as those vertebrae between the skull and the first rib-bearing vertebrae (Cerbus et al., 2024). The length of the neck is considered to be expressed in terms of the number and size of the cervical vertebrae that make up the neck. The vertebrae in the neck and chest can be anatomically distinguished as cervical vertebrae (without ribs) and thoracic vertebrae (with ribs). Although there is a diversity of species, the count of the cervical vertebrae has been fixed almost universally at seven in mammals (Buchholtz et al., 2012). However, birds show great morphological diversity in the neck, with the number of cervical vertebrae varying from 10 to 26, and chickens have been reported to have 14 cervical vertebrae and seven ribs (Marek et al., 2021). To date, morphometric comparisons in CT images of the Tosa-jidori, Chabo, and Minohikichabo have found diversity in the number of caudal vertebrae (Kondoh et al., 2022). However, almost no investigation of the skeleton, including the neck and chest, has been conducted in the Japanese indigenous chickens. Therefore, this study aims to find morphological differences among the four Japanese indigenous chicken breeds, Tosa-jidori, Ko-shamo, Minohikichabo, and Chabo, using skeletal specimens.

## Materials and methods

### Animals

Seven-week-old individuals of Tosa-jidori (male n = 11, female n = 9), Ko-shamo (male n = 10, female n = 11), Minohikichabo (male n = 28, female n = 26), and Chabo (male n = 15, female n = 11) were used. All individuals were reared in an ad libitum condition for feed and water in the poultry farm at Obihiro University of Agriculture and Veterinary Medicine, Japan. The chickens were slaughtered by cutting the carotid arteries, and the carcasses were frozen and stored at −30°C until use. The experimental protocol for this study was approved by the Animal Care and Use Committee of Obihiro University of Agriculture and Veterinary Medicine (approval numbers 23-29 and 24-45).

Adult (> 40 weeks of age) individuals of Tosa-jidori (male n = 8, female n = 9), Ko-shamo (male n = 14, female n = 26), Minohikichabo (male n = 16, female n = 22), and Chabo (male n = 25, female n = 13) were used. The adult stage samples include both dead and slaughtered individuals. The samples were stored at −30°C until use.

### Preparation of skeletal specimens and assessment of morphology

Before the sampling at 7 weeks of age and the adult stage, body weight and shank (leg) length were measured. Computed tomography (CT) images of the whole body were collected using an Aquilion TSX-201A scanner (Canon Medical Systems Co. Ltd., Otawara, Japan) under the conditions of 120 kV, 250 mA, and 0.5 mm slice thickness. CT data (DICOM format) were checked using RadiAnt DICOM Viewer (Medixant, 2023). Skeletal structures were visualized using the Angio and Bones B/W modes of a 3D Volume Rendering function (Kondoh et al., 2022).

Skeletal specimens at 7 weeks of age in Tosa-jidori (male n = 8, female n = 8), Ko-shamo (male n = 6, female n = 9), Minohikichabo (male n = 6, female n = 5), and Chabo (male n = 10, female n = 9) were prepared from the slaughtered chickens to verify bone morphology. The whole-body skin from the thawed carcass is removed. Bones such as the shoulder blades and legs are removed to assess the neck and chest parts. After boiling the meat using hot water, the meat is removed from the bones using tweezers. Finally, the bones are bleached by 10% hydrogen peroxide solution (H_2_O_2_).

Visual comparisons were conducted across the entire skeletal specimen. In addition, the presence or absence of ribs in the vertebrae was assessed to determine the cervical (without rib) and thoracic (with rib) vertebrae. The prepared skeletal specimens were then used to measure neck length and body length at 7 weeks of age. Neck length was defined as the length from the second to the last cervical vertebrae. Body length was defined as the length from the second cervical vertebra to the last caudal vertebra. The neck length and body length were measured using a tape measure. These traits were used to calculate the percentage of neck length in body length. Adult (> 40 weeks of age) samples were evaluated by CT images.

### Statistical analysis

The main effects of breed and sex and their interaction effects on body weight, shank length, neck length, and percentage of neck length in body length were tested by two-way analysis of variance (ANOVA). If a significant difference (*P* < 0.05) is found, a multiple comparison test was performed using Tukey’s HSD test.

Since variation in the number of ribs was found in this study, the proportion of occurrences of the number of ribs in each breed was tested using Fisher’s exact test. Data was shown by mean ± SD. All analyses were performed using R (R Core Team, 2025) and RStudio (RStudio Team, 2025).

## Results

### Growth traits at 7 weeks of age

Body weights at 7 weeks of age were 280.1 ± 24.2 g for male Tosa-jidori and 231.2 ± 17.5 g for female Tosa-jidori, 269.4 ± 49.6 g for male Ko-shamo, 188.3 ± 40.2 g for female Ko-shamo, 240.5 ± 23.4 g for male Minohikichabo, 198.2 ± 31.9 g for female Minohikichabo, and 240.8 ± 32.0 g for male Chabo, 206.1 ± 21.9 g for female Chabo (Table 1). Two-way ANOVA showed significant main effects of breed (F_3,__113_ = 7.107, *p* = 0.0002) and sex (F_1,__113_ = 70.044, *p* = 1.7e-13), but no interaction effect of breed and sex (F_3,__113_ = 2.408, *p* = 0.071) in the body weight. Tosa-jidori indicated significantly heavier body weight than the other breeds at 7 weeks of age (*p* < 0.05). Males were significantly heavier than females (*p* < 0.001).

**Table 1:** Growth traits at 7 weeks of age in 4 Japanese indigenous chicken breeds.

Trait^1^	Tosa-jidori (TSJ)		Ko-shamo (KSM)		Minohikichabo (MHC)		Chabo (CHB)		Two-way ANOVA^2^		
	M (*n* = 11)	F (*n* = 9)	M (*n* = 10)	F (*n* = 11)	M (*n* = 28)	F (*n* = 26)	M (*n* = 15)	F (*n* = 11)	Breed	Sex	B*S
BW	280.1 ± 24.2	231.2 ± 17.5	269.4 ± 49.6	188.3 ± 40.2	240.5 ± 23.4	198.2 ± 31.9	240.8 ± 32.0	206.1 ± 21.9	***	***	ns
SL	47.4 ± 2.1	43.3 ± 1.6	46.9 ± 4.3	39.6 ± 4.2	39.9 ± 4.2	34.8 ± 4.5	38.3 ± 3.3	35.8 ± 1.8	***	***	ns

1 BW: body weight at 7 weeks of age, SL: shank length at 7 weeks of age.

2 ***p < 0.001; **p < 0.01; *p < 0.05

ns *p* > 0.05. B*S: interaction effects of breed and sex.

Shank lengths at 7 weeks of age were 47.4 ± 2.1 mm for male Tosa-jidori and 43.3 ± 1.6 mm for female Tosa-jidori, 46.9 ± 4.3 mm for male Ko-shamo, 39.6 ± 4.2 mm for female Ko-shamo, 39.9 ± 4.2 mm for male Minohikichabo, 34.8 ± 4.5 mm for female Minohikichabo, and 38.3 ± 3.3 mm for male Chabo, 35.8 ± 1.8 mm for female Chabo (Table 1). In shank length, two-way ANOVA revealed significant main effects of breed (F_3,__113_ = 30.931, *p* = 1.1e-14) and sex (F_1,__113_ = 46.058, *p* = 5.6e-10), but not the interaction effect of breed and sex (F_3,__113_ = 1.562, *p* = 0.202). Compared with the Chabo and Minohikichabo, the Tosa-jidori and Ko-shamo breeds exhibited significantly longer shank lengths (*p* < 0.001). Males have significantly longer shank lengths than females (*p* < 0.001).

### Skeletal traits at 7 weeks of age

The lengths of cervical vertebrae at 7 weeks of age were 6.6 ± 0.4 cm for male Tosa-jidori, 6.1 ± 0.2 cm for the female Tosa-jidori, 6.3 ± 0.2 cm for male Ko-shamo, 5.7 ± 0.5 cm for female Ko-shamo, 5.7 ± 0.6 cm for male Minohikichabo, 5.6 ± 0.4 cm for female Minohikichabo, and 5.4 ± 0.5 cm for male Chabo, 5.4 ± 0.3 cm for female Chabo (Table 2). Two-way ANOVA showed significant main effects of breed (F_3,__53_ = 13.946, *p* = 8.0e-7) and sex (F_1,__53_ = 5.937, *p* = 0.018), but not the interaction effect of breed and sex (F_3,__53_ = 2.042, *p* = 0.119). Neck length of the Tosa-jidori was significantly longer than that of the Minohikichabo and Chabo (*p* < 0.001), although neck length of the Chabo was significantly shorter than that of the Ko-shamo (*p* < 0.01). Males exhibited significantly longer necks than females (*p* < 0.05).

**Table 2:** Skeletal traits at 7 weeks of age in 4 Japanese indigenous chicken breeds.

Trait^1^	Tosa-jidori (TSJ)		Ko-shamo (KSM)		Minohikichabo (MHC)		Chabo (CHB)		Two-way ANOVA^2^		
	M (*n* = 8)	F (*n* = 8)	M (*n* = 6)	F (*n* = 9)	M (*n* = 6)	F (*n* = 5)	M (*n* = 10)	F (*n* = 9)	Breed	Sex	B*S
Neck length	6.6 ± 0.4	6.1 ± 0.2	6.3 ± 0.2	5.7 ± 0.5	5.7 ± 0.6	5.6 ± 0.4	5.4 ± 0.5	5.4 ± 0.3	***	*	ns
Body length	15.8 ± 1.1	14.9 ± 0.5	14.9 ± 0.7	13.4 ± 0.9	13.7 ± 1.2	13.3 ± 1.0	13.9 ± 1.1	13.7 ± 0.7	***	**	ns

1 Neck length: length of cervical vertebrae at 7 weeks of age, Body length: length from cervical vertebrae to caudal vertebrae at 7 weeks of age.

2 ***p < 0.001; **p < 0.01; *p < 0.05.

ns *p* > 0.05. B*S: interaction effects of breed and sex.

Body lengths at 7 weeks of age were 15.8 ± 1.1 cm for male Tosa-jidori, 14.9 ± 0.5 cm for female Tosa-jidori, 14.9 ± 0.7 cm for male Ko-shamo, 13.4 ± 0.9 cm for female Ko-shamo, 13.7 ± 1.2 cm for male Minohikichabo, 13.3 ± 1.0 cm for female Minohikichabo, and 13.9 ± 1.1 cm for male Chabo, 13.7 ± 0.7 cm for female Chabo (Table 2). Two-way ANOVA revealed significant main effects of breed (F_3,__53_ = 10.133, *p* = 2.2e-5) and sex (F_1,__53_ = 8.139, *p* = 0.0062), but not interaction effect (F_3,__53_ = 1.239, *p* = 0.305). The body length of Tosa-jidori was significantly longer than that of the other breeds (*p* < 0.01). Males had significantly longer body length than females (*p* < 0.01).

### Ratio of neck length to body length (%) at 7 weeks of age

**Fig. 2:**
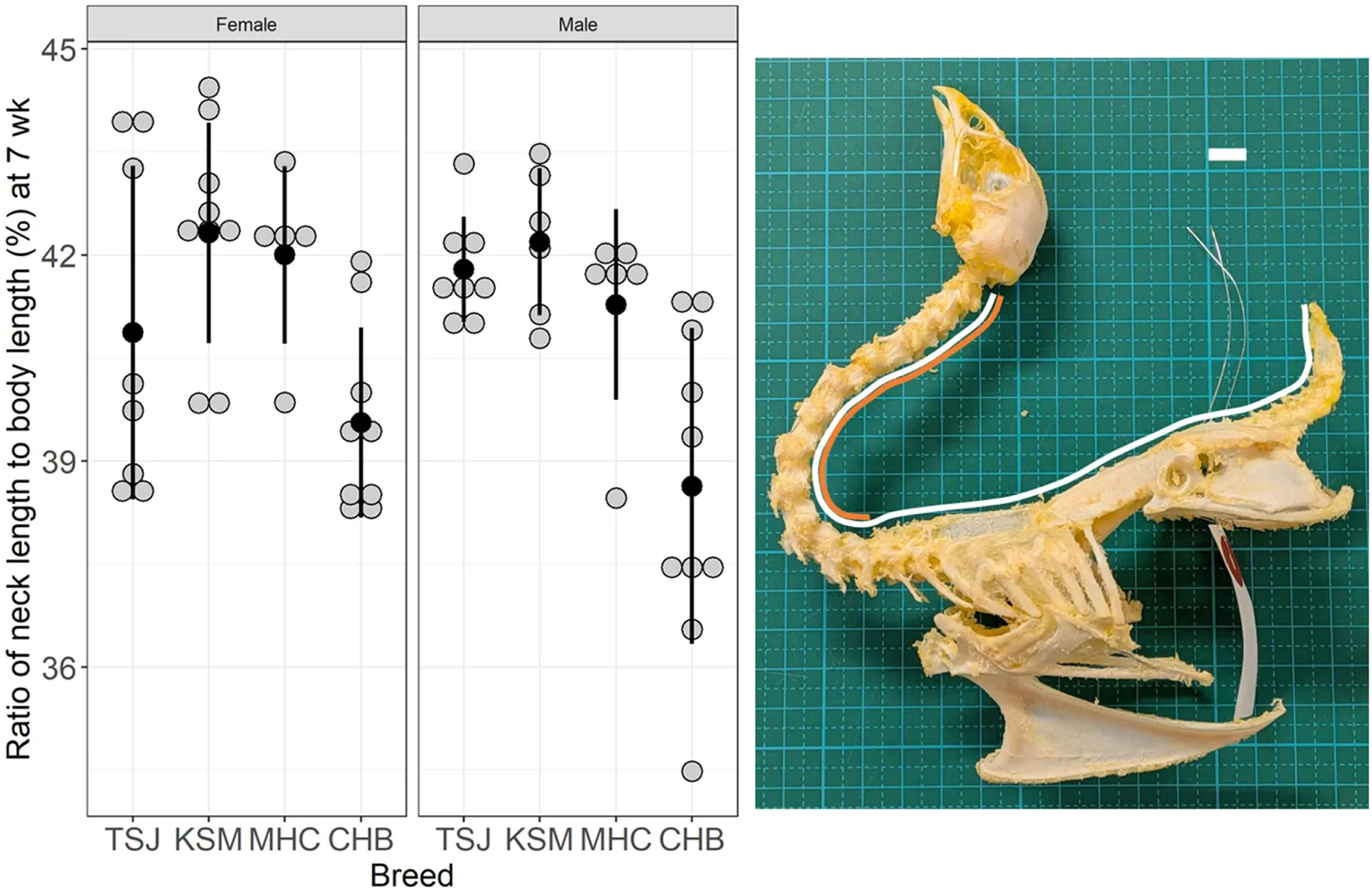
Ratio of neck length to body length of four Japanese indigenous breeds at 7 weeks of age. Two-way ANOVA reveals a significant breed effect (P = 5.5e-6). Chabo has a significantly lower ratio than the other breeds. Black dot and bar indicate mean and SD. Gray dots show individual values. TSJ: Tosa-jidori, KSM: Ko-shamo, MHC: Minohikichabo, CHB: Chabo. The right image indicates a skeletal specimen. White and orange curves indicate the body and neck lengths, respectively. The length of the white bar indicates 1 cm.

The ratio of neck length to body length (%) was 41.8 ± 0.7% for male Tosa-jidori, 40.9 ± 2.3% for female Tosa-jidori, 42.2 ± 1.0% for male Ko-shamo, 42.3 ± 1.5% for female Ko-shamo, 41.3 ± 1.3% for male Minohikichabo, 42.0 ± 1.2% for female Minohikichabo, and 38.6 ± 2.2% for male Chabo, 39.6 ± 1.3% for female Chabo (Fig. 2). A significant main effect of breed (F_3,__53_ = 11.688, *p* = 5.5e-6) was found by two-way ANOVA, but no main effect of sex (F_1,__53_ = 0.229, *p* = 0.634) and interaction effect (F_3,__53_ = 0.969, *P* = 0.414). Chabo had a significantly lower ratio of neck length to body length than the other breeds (*p* < 0.01). There was no difference between male and female (*p* = 0.636).

### Correlation between neck length and leg length

**Fig. 3:**
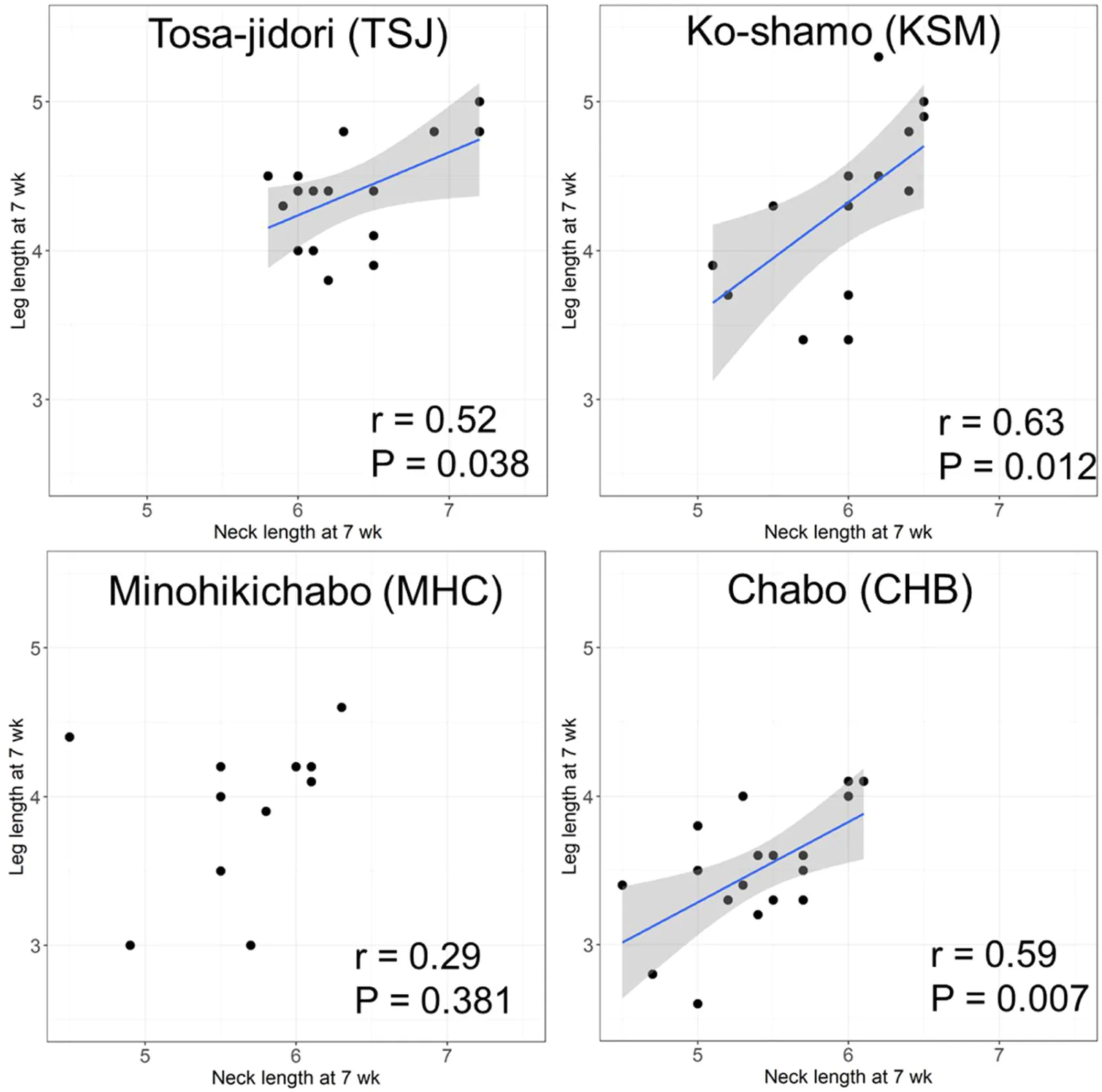
Scatter plots of neck length and leg length in four Japanese indigenous breeds at 7 weeks of age. There are significant positive correlations between neck length and leg length in Tosa-jidori (*r* = 0.52, *p* = 0.038), Ko-shamo (*r* = 0.63, *p* = 0.012), and Chabo (*r* = 0.59, *p* = 0.007). No correlation is found in Minohikichabo (*r* = 0.29, *p* = 0.381).

To see the relationship between the two phenotypes of neck length and leg length at 7 weeks of age, scatter plots were generated in the four breeds (Fig. 3). Phenotypic correlation coefficients for neck length and leg length were *r* = 0.52 (*p* < 0.05) for Tosa-jidori, *r* = 0.63 (*p* < 0.05) for Ko-shamo, *r* = 0.29 (*p* = 0.381) for Minohikichabo, and *r* = 0.59 (*p* < 0.05) for Chabo. Positive correlations were observed in Tosa-jidori, Ko-shamo, and Chabo, although no correlation was found in Minohikichabo.

### Number of ribs

**Fig. 4:**
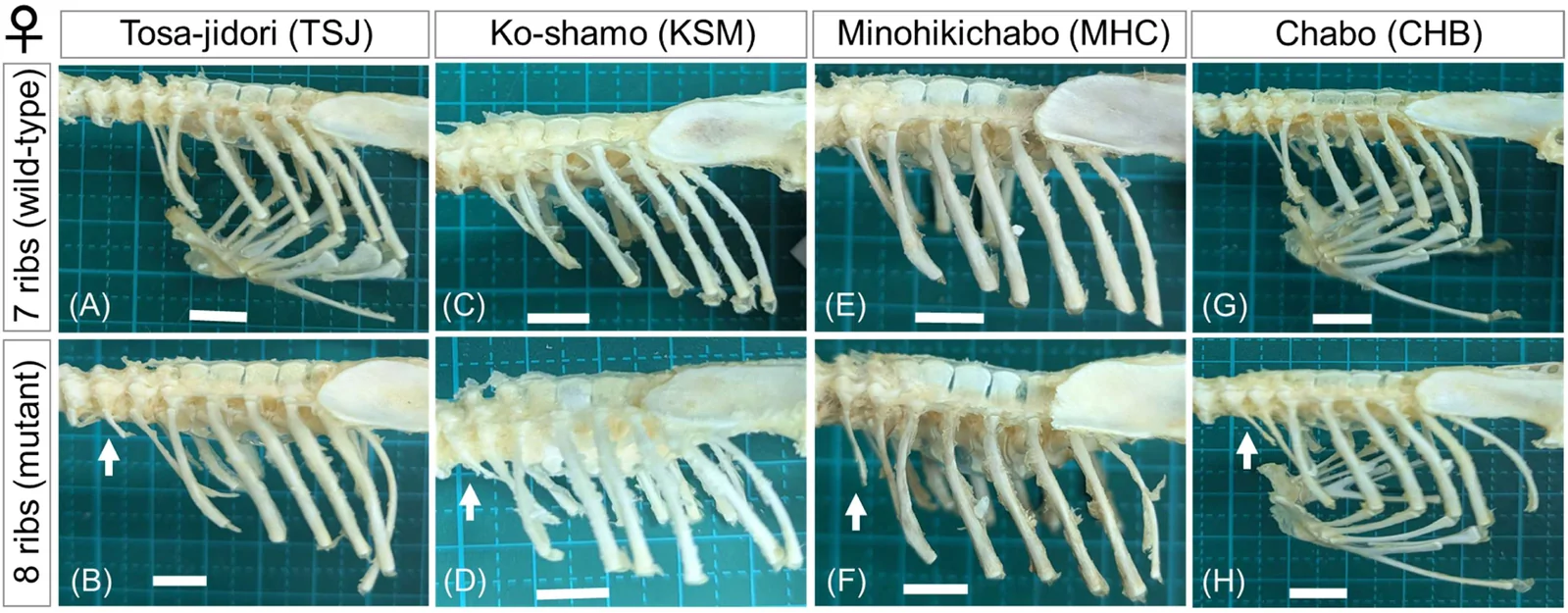
Variation of rib number of females in the four Japanese indigenous chicken breeds at 7 weeks of age. (A) 7-ribs Tosa-jidori. (B) 8-ribs Tosa-jidori. (C) 7-ribs Ko-shamo. (D) 8-ribs Ko-shamo. (E) 7-ribs Minohikichabo. (F) 8-ribs Minohikichabo. (G) 7-ribs Chabo. (H) 8-ribs Chabo. The length of the white bar indicates 1 cm. The white arrow indicates an additional rib.

**Fig. 5:**
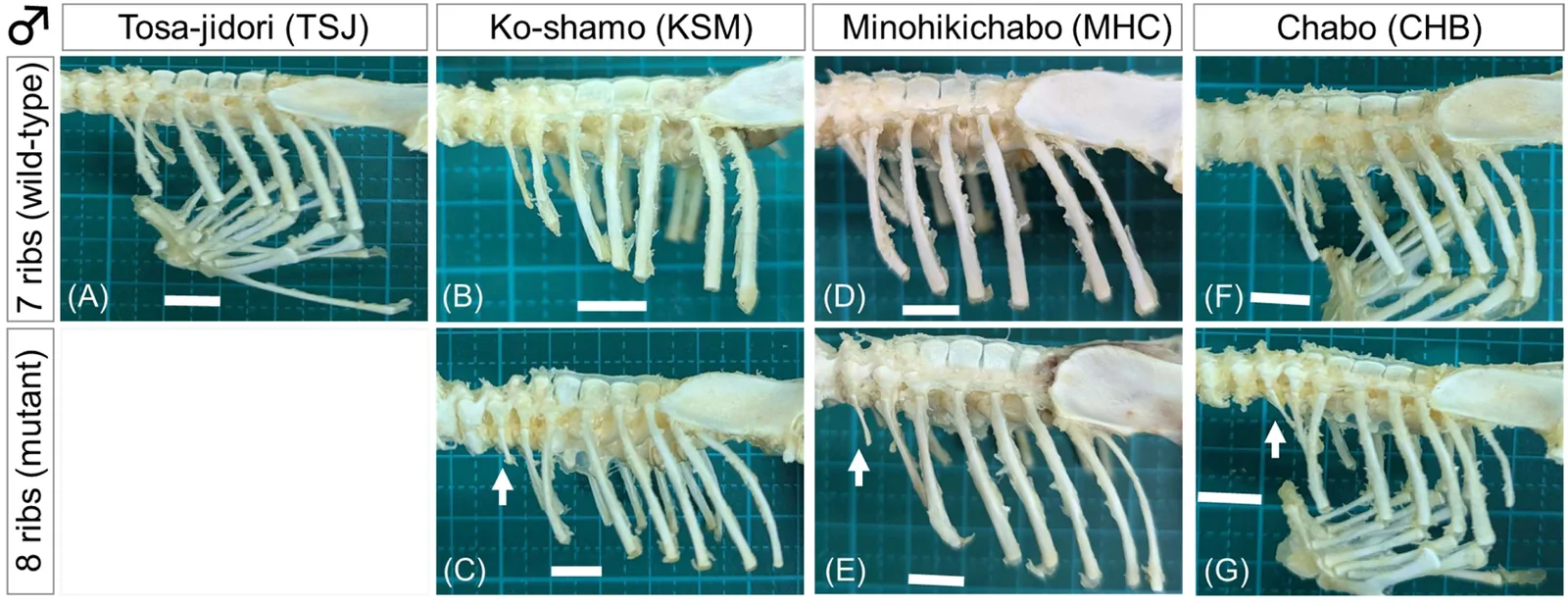
Variation of rib number of males in the four Japanese indigenous chicken breeds at 7 weeks of age. (A) 7-ribs Tosa-jidori. (B) 7-ribs Ko-shamo. (C) 8-ribs Ko-shamo. (D) 7-ribs Minohikichabo. (E) 8-ribs Minohikichabo. (F) 7-ribs Chabo. (G) 8-ribs Chabo. The length of the white bar indicates 1 cm. The white arrow indicates an additional rib.

The vertebrae are divided into cervical, thoracic, lumbar, sacral, and caudal vertebrae. The cervical, thoracic, and lumbar vertebrae can be distinguished by the presence or absence of ribs due to the similar shape of the vertebrae. Chickens are reported to have seven ribs (Marek et al., 2021). Our study using skeletal specimens at 7 weeks of age identified individuals with seven ribs (as previously reported) and novel individuals with eight ribs in females (Fig. 4) and males (Fig. 5).

The contingency table showed the number of individuals with seven ribs and eight ribs at 7 weeks of age in 4 breeds (Table 3). Observations of skeletal specimens from 16 Tosa-jidori (female n = 8 and male n = 8) revealed that one female and 0 male had eight ribs. In the skeletal specimens of 15 Ko-shamo (n = 9 females and n = 6 males), two females and one male had eight ribs. In skeletal specimens of 11 Minohikichabo individuals (n = 5 females and n = 6 males), three females and four males had eight ribs. Observations of skeletal specimens from 19 Chabo (female n = 9 and male n = 10) revealed that six females and seven males had eight ribs. The rib number of the four breeds showed significant differences in proportions (*p* < 0.001, Fisher’s exact test). Minohikichabo and Chabo had a higher proportion of individuals with eight ribs (63.6% and 68.4%), while the Tosa-jidori and Ko-shamo had a lower proportion of individuals with eight ribs (6.3% and 20.0%).

**Table 3:** Contingency table for rib number (7 or 8) at 7 weeks of age in 4 breeds.

Breed	No. of rib = 7 (R7)			No. of rib = 8 (R8)		
	Total (%)	♀	♂	Total (%)	♀	♂
Tosa-jidori (TSJ)	15 (93.8%)	7	8	1 (6.3%)	1	0
Ko-shamo (KSM)	12 (80.0%)	7	5	3 (20.0%)	2	1
Minohikichabo (MHC)	4 (36.4%)	2	2	7 (63.6%)	3	4
Chabo (CHB)	6 (31.6%)	3	3	13 (68.4%)	6	7

Significant differences in proportions (*p* = 0.00014, Fisher’s exact test).

**Fig. 6:**
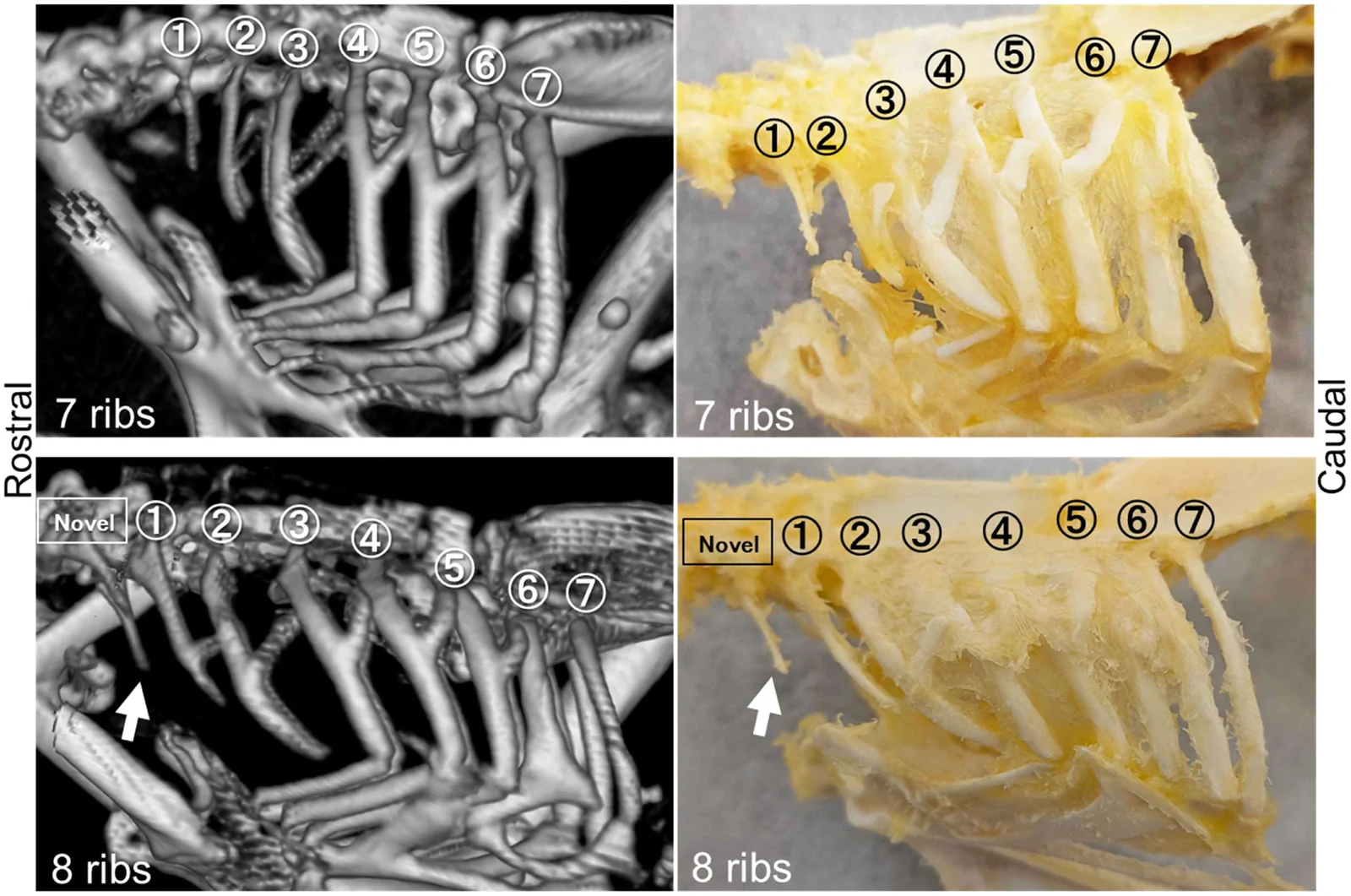
Comparison with the CT images and skeletal specimens in the four Japanese indigenous chicken breeds at the adult stage. The upper and lower panels indicated an individual with 7 ribs (wild-type) and 8 ribs (mutant), respectively. The left panel (CT image) and right panel (skeletal specimen) represent an identical sample. The white arrow indicates a novel additional rib.

To verify whether the novel finding is consistent with observations at the mature stage, the CT images of adult (> 40 weeks of age) stage samples were evaluated. CT images of samples at the adult stage indicated clear rib images, which are consistent with the skeletal specimens (Fig. 6). Therefore, the evaluation for adult stage samples was conducted using the CT images. As shown in the contingency table (Table 4), we could observe individuals of seven ribs and eight ribs at the adult stage in 4 breeds. Observations from 17 Tosa-jidori (female n = 9 and male n = 8) revealed that two females and one male had eight ribs. In the 40 Ko-shamo (n = 26 females and n = 14 males), eight females, and three males had eight ribs. In the CT images of 38 Minohikichabo (n = 22 females and n = 16 males), 11 females and nine males had eight ribs. Observations of CT images from 38 Chabo (female n = 13 and male n = 25) revealed that six females and 11 males had eight ribs. Fisher’s exact test revealed significant differences in proportions among breeds (*p* < 0.05). Minohikichabo and Chabo had a higher proportion of individuals with eight ribs (52.6% and 44.7%), although the Tosa-jidori and Ko-shamo had a lower proportion of individuals with eight ribs (17.6% and 27.5%).

**Table 4:** Contingency table for rib number (7 or 8) at the adult stage in 4 breeds.

Breed	No. of rib = 7 (R7)			No. of rib = 8 (R8)		
	Total (%)	♀	♂	Total (%)	♀	♂
Tosa-jidori (TSJ)	14 (82.4%)	7	7	3 (17.6%)	2	1
Ko-shamo (KSM)	29 (72.5%)	18	11	11 (27.5%)	8	3
Minohikichabo (MHC)	18 (47.4%)	11	7	20 (52.6%)	11	9
Chabo (CHB)	21 (55.3%)	7	14	17 (44.7%)	6	11

Significant differences in proportions (*p* = 0.02936, Fisher’s exact test).

**Fig. 7:**
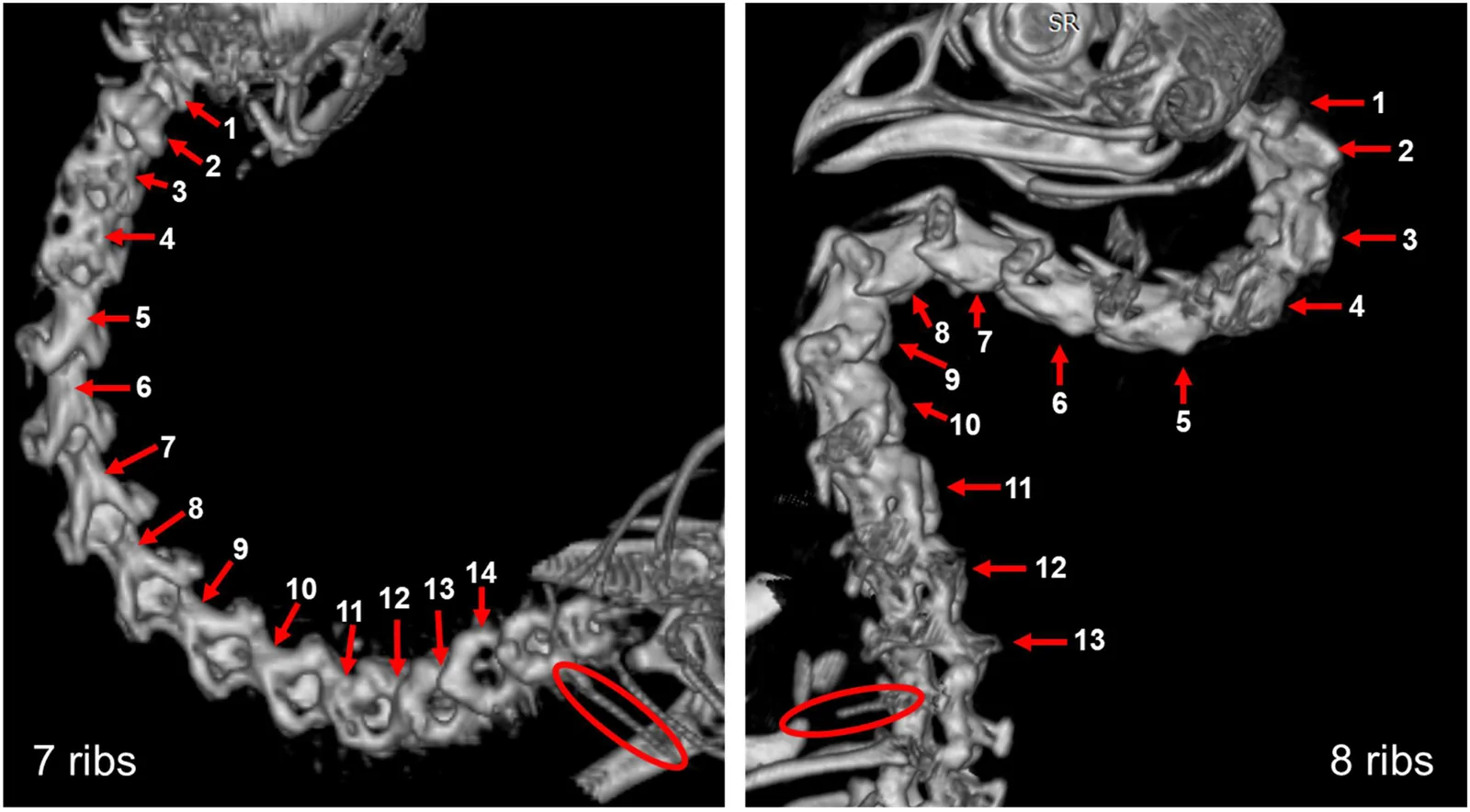
Number of vertebrae of chickens with 7 ribs (wild-type) and 8 ribs (novel mutant) at the adult stage. A wild-type individual has 14 cervical vertebrae and 7 thoracic vertebrae (rib-bearing vertebrae). The novel individual has 13 cervical vertebrae and 8 thoracic vertebrae. The ellipse represents the first rib.

Fig. 7 shows the number of cervical vertebrae of individuals with seven ribs and eight ribs at the adult stage. Although a wild-type individual (seven ribs) has 14 cervical vertebrae and 7 thoracic vertebrae (rib-bearing vertebrae), the present novel mutant individual (eight ribs) has 13 cervical vertebrae and 8 thoracic vertebrae. Since the total number of cervical and thoracic vertebrae is the same in seven- and eight-rib cases, the present eight-rib condition should be a novel homeotic mutation.

## Discussion

This study compared morphological traits using skeletal specimens and CT images of four Japanese indigenous chicken breeds at 7 weeks of age and the adult stage. The Tosa-jidori had significantly higher body weight and body length than the others at 7 weeks of age. Shank lengths of the Tosa-jidori and Ko-shamo were significantly longer than those of the Minohikichabo and Chabo. The neck length of the Tosa-jidori was significantly longer than that of Chabo. The ratio of neck length to body length of the Chabo was significantly lower than that of the others. Positive correlations between neck length and leg length were observed, except for the Minohikichabo. Our observation identified novel mutants (individuals with eight ribs) across the four Japanese indigenous breeds. Significant breed differences were observed in the ratio of wild-type (seven ribs) to mutant (eight ribs) at both 7 weeks of age and the adult stage. The wild-type was predominant in Tosa-jidori and Ko-shamo, whereas the mutant was the majority or roughly half-and-half in Minohikichabo and Chabo. Based on the total number of cervical and thoracic vertebrae, the present eight ribs should represent a novel homeotic mutation. This study described the morphological characteristics of four Japanese indigenous chicken breeds and documented novel variation of rib number within an avian species, chicken (*Gallus gallus domesticus*).

Growth and morphological traits of Tosa-jidori, Minohikichabo, and Chabo have been investigated from 1 to 36 weeks of age (Ono et al., 2022). The present results on body weights and shank lengths at 7 weeks of age were similar to those of the previous report (Ono et al., 2022). This study allowed for the addition of new data for the Ko-shamo, which was found to have significantly smaller body weight and shorter body length than the Tosa-jidori at 7 weeks of age. However, the shank length and neck length of the Tosa-jidori and Ko-shamo were similar to each other. Ono et al. (2022) indicated that skeletal growth (shank length) is faster than body size growth (body weight), suggesting that the approximate stages at which the three breeds reached a plateau were around 13 and 24 weeks of age for shank length and body weight, respectively. It is therefore likely that the growth speed of the Tosa-jidori is faster than that of the Ko-shamo at 7 weeks of age in body weight and body length (cervical to caudal vertebra), but not in shank length (tarsometatarsus) and neck length (cervical vertebrae). Although shank length has traditionally been used as an indicator of skeletal growth (Lerner, 1937; Edward and Morley, 1946; Goto et al., 2019), it has become clear that the growth rates of bones in the other body parts may vary. Kondoh et al. (2022) clearly indicated that the breed-specific angles of the pygostyle (caudal bone) in three Japanese indigenous breeds support the breed-specific angles of the tail feathers, which contribute to the breed-specific visual appearance of the caudal part. In the future, age-dependent changes in growth and whole-body skeletal traits from chick to adult should be monitored on the four Japanese chicken breeds to gain a deeper understanding of the breed-specific morphological characteristics.

Our hypothesis at the beginning of the study was that the neck length of each breed differs, especially the Ko-shamo having a long neck. The present study revealed that there was no difference among the Ko-shamo, Minohikichabo, and Tosa-jidori in the ratio of neck length to body length at 7 weeks of age. This suggested that the visual appearance of a longer neck may be due to the feathering around the neck. The Ko-shamo may have significantly shorter and fewer feathers around the neck to chest compared to the other breeds, which may influence the visual impression of body shape. In addition, the Chabo, one of the most popular chicken breeds among fanciers in Japan, had a significantly lower ratio of neck length to body length than the others. The previous skeletal comparison of six Japanese indigenous breeds, including the Chabo (Kudo et al., 2016; 2017), revealed that the Chabo has a small skull, large eye sockets, short forelimb bones, and wide articulation, which is likely to create a round and soft body silhouette. The low neck-to-body length ratio in Chabo identified in this study may also contribute to the overall rounded appearance of the body shape. It is speculated that the Chabo may have been artificially selected as a pet to preserve its distinctive characteristics.

Although the neck length tends to decrease with increasing body size in mammals, it is known that the length of the cervical vertebral column increases as body size increases (positive correlation, *r* = 0.91) across 68 bird species, including sparrows, crows, ostriches, and penguins (Bohmer et al., 2019). The correlated evolution of neck length and total leg length in extant birds (Bohmer et al., 2019) was commonly detected in the Tosa-jidori, Ko-shamo, and Chabo, but not in Minohikichabo. Compared with wild birds, Japanese indigenous chicken breeds seem to have been shaped by long-term artificial selection to alter body shape. The Chabo and Minohikichabo have generally shorter legs on average than the Tosa-jidori and Ko-shamo. However, the Minohikichabo showed greater variation in leg length (SD = 4.2 and 4.5 for males and females, respectively) than Chabo (SD = 3.3 and 1.8 for males and females, respectively). Moreover, the individuals with unique body proportions (shorter neck but longer legs) could be observed in the scatter plot of Minohikichabo. Thus, artificial selection may not have been completed to produce shorter legs, and then variable body proportions may have been retained in the Minohikichabo population.

Khabyuk et al. (2022) have reported that the occurrence of an eighth rib in 8-day embryos of White Leghorn. Their observation using the chicken embryo from HH-stage 24 (embryonic day 4) up to HH-stage 37 (embryonic day 11) identified the variable and temporal presence of the eighth rib in approximately 40% of cases and the additional rib observed on the lumbar side (Khabyuk et al., 2022). The present study confirmed the stable presence of eight ribs and the additional rib observed on the cervical side in postnatal growing and mature stages. These results suggested that our cervical-side rib in Japanese indigenous breeds has a different origin from the lumbar-side rib in White Leghorn embryo. As the next experiments, investigations are needed to answer the questions: at which embryonic stages does our homeotic mutant occur, whether the mutant is inherited, what is the mode of inheritance, and which genes regulate its occurrence. From a body of evidence (Wellik, 2007; Vinagre et al., 2010; Buchholtz et al., 2012; Cerbus et al., 2024), *HOX* genes are likely to be strong candidates for altering axial skeleton patterning. In addition, European commercial pig breeds have 21–23 vertebrae, although wild boars (the wild ancestor of pigs) have 19 vertebrae. Swine *NR6A1* and *VRTN* have been reported as strong candidates for adding thoracic segments, which will alter the expression patterns of *Hox* genes (Mikawa et al., 2007; 2011). Moreover, Wnt, FGF, and Notch signaling pathways are also strong candidates, as they regulate body axis formation and patterning (Bénazéraf and Pourquié, 2013). Therefore, we need to answer how these candidate genes affect the occurrence of our cervical-side rib. In the near future, these remaining research questions should be confirmed.

This study confirmed that many individuals with eight ribs exist in Japanese indigenous chickens. A wide variety of morphological characteristics is observed in Tosa-jidori (wild-type body shape), Ko-shamo (Malay-type body shape), Minohikichabo (wild-type body shape with rich tail feathers), and Chabo (ball-like body shape), as supported by their separated phylogenetic positions (Osman et al., 2006). From approximately 45 Japanese indigenous breeds (Tsudzuki, 2003; Matsuda et al., 2025), we focused on the four Japanese indigenous breeds, which are all categorized as miniature breeds (dwarf chickens). Therefore, the eight-rib mutant may be derived from a dwarf-related alteration. In the future, several Japanese indigenous breeds that are related to the four breeds but not dwarf (e.g., Shokoku, Gifu-jidori, Oh-shamo, and Yakido) should be investigated to determine whether individuals with eight ribs are observed in dwarf chickens only or not. If the exploration is expanded to include not only Japanese indigenous breeds but also Asian breeds and red jungle fowl, the origin of the mutant will be revealed.

Our cervical-side rib phenotype in chickens is similar to examples in human cases, the so-called cervical rib. Although humans have seven cervical vertebrae, the case has an incomplete rib at the seventh cervical vertebra. The cervical rib is found in approximately 0.5% of the total human population and is highly observed in cases of thoracic outlet syndrome (Ghany et al., 2017). Moreover, Cuxart-Erruz et al. (2024) have noted that, based on lines of evidence, including the incidence of abnormal cervical vertebral numbers in mammoths (*Mammuthus primigenius*) and woolly rhinoceroses (*Coelodonta antiquitatis*) shortly before their extinction, cervical rib incidence may be a good predictor of extinction risk in inbred mammalian populations. If it becomes clear that cases of cervical rib in Japanese indigenous chickens are caused by genetic variants similar to those in human cervical ribs and other mammals at risk of extinction, this could lead to a better understanding of the mechanisms. This suggests that the Japanese indigenous chicken has high potential as a unique model organism in morphology and genetic resources (Goto and Tsudzuki, 2017; Cheng and Burt, 2018).

In this study, skeletal specimens of four Japanese chicken breeds, Tosa-jidori, Ko-shamo, Minohikichabo, and Chabo, were prepared with the aim of finding breed differences in morphology. The morphological characteristics of the four Japanese chicken breeds at seven weeks of age and the adult stage were clarified, and the diversity in the number of ribs in chickens was found for the first time. In the future, our genetic analysis using intercross populations, including morphological comparisons using skeletal specimens in embryos and postnatal chickens, will reveal the genetic basis controlling the homeotic mutation in rib number.

## Disclosures

The authors declare no conflict of interest.

## Author Contributions

**Yume Okada** performed the animal experiments and investigation, and drafted the original manuscript and data visualization. **Yuma Nishida, Dipson Gyawali, Prudence Nyirimana, and Jumpei Tomiyasu** participated in the animal experiments and sample processing. **Daisuke Kondoh, Ryota Iwasaki, and Masaki Eda** participated in data curation and contributed to the development of experimental protocols. **Tatsuhiko Goto** conceived the study, designed and supervised the project, secured funding, contributed to data interpretation, drafted the original manuscript and data visualization, and reviewed and revised the manuscript. All authors have read and approved the final version and agree to be accountable for all aspects of the work.
